# A Transfer Entropy Approach for the Assessment of the Impact of Inspiratory Muscle Training on the Cardiorespiratory Coupling of Amateur Cyclists

**DOI:** 10.3389/fphys.2020.00134

**Published:** 2020-02-25

**Authors:** Raphael Martins de Abreu, Aparecida Maria Catai, Beatrice Cairo, Patricia Rehder-Santos, Claudio Donisete da Silva, Étore De Favari Signini, Camila Akemi Sakaguchi, Alberto Porta

**Affiliations:** ^1^Department of Physical Therapy, Federal University of São Carlos, São Carlos, Brazil; ^2^Department of Biomedical Sciences for Health, University of Milan, Milan, Italy; ^3^Department of Cardiothoracic – Vascular Anesthesia and Intensive Care, IRCCS Policlinico San Donato, Milan, Italy

**Keywords:** multivariate linear regression model, sport medicine, breathing exercise, heart rate variability, complexity, autonomic nervous system, cardiac control, baroreflex

## Abstract

The strength of cardiorespiratory interactions diminishes with age. Physical exercise can reduce the rate of this trend. Inspiratory muscle training (IMT) is a technique capable of improving cardiorespiratory interactions. This study evaluates the effect of IMT on cardiorespiratory coupling in amateur cyclists. Thirty male young healthy cyclists underwent a sham IMT of very low intensity (SHAM, *n* = 9), an IMT of moderate intensity at 60% of the maximal inspiratory pressure (MIP60, *n* = 10) and an IMT of high intensity at the critical inspiratory pressure (CIP, *n* = 11). Electrocardiogram, non-invasive arterial pressure, and thoracic respiratory movement (RM) were recorded before (PRE) and after (POST) training at rest in supine position (REST) and during active standing (STAND). The beat-to-beat series of heart period (HP) and systolic arterial pressure (SAP) were analyzed with the RM signal via a traditional non-causal approach, such as squared coherence function, and via a causal model-based transfer entropy (TE) approach. Cardiorespiratory coupling was quantified via the HP-RM squared coherence at the respiratory rate (*K*^2^_HP–R__M_), the unconditioned TE from RM to HP (TE_R__M__→__HP_) and the TE from RM to HP conditioned on SAP (TE_R__M__→__HP| SAP_). In PRE condition we found that STAND led to a decrease of TE_R__M__→__HP| SAP_. After SHAM and CIP training this tendency was confirmed, while MIP60 inverted it by empowering cardiorespiratory coupling. This behavior was observed in presence of unvaried SAP mean and with usual responses of the baroreflex control and HP mean to STAND. TE_R__M__→__HP_ and *K*^2^_HP–__RM_ were not able to detect the post-training increase of cardiorespiratory coupling strength during STAND, thus suggesting that conditioning out SAP is important for the assessment of cardiorespiratory interactions. Since the usual response of HP mean, SAP mean and baroreflex sensitivity to postural stressor were observed after MIP60 training, we conclude that the post-training increase of cardiorespiratory coupling during STAND in MIP60 group might be the genuine effect of some rearrangements at the level of central respiratory network and its interactions with sympathetic drive and vagal activity.

## Introduction

In the field of the analysis of spontaneous fluctuations of heart period (HP) with the term *cardiorespiratory coupling* (CRC) is intended the set of mechanisms responsible for a variable quote of HP variability (HPV) driven by respiration. For example, respiratory sinus arrhythmia (RSA) (Hirsch and Bishop, 1981) is considered to be, at least partially, the genuine consequence of the activity of respiratory centers modulating vagal motoneuron responsiveness and activity (Eckberg and Karemaker, 2009). The abovementioned definition has two important consequences over CRC assessment: (i) both HPV and respiration need to be acquired, namely at least a bivariate analysis framework should be arranged (Bracic Lotric and Stefanovska, 2000; Bartsch et al., 2007; Porta et al., 2012, 2015; Penzel et al., 2016; Mazzucco et al., 2017); (ii) directionality of the interactions (i.e. from respiration to HPV) must be taken into account, thus restricting signal processing methods suitable to be exploited to those belonging to the causal class (Porta et al., 2012, 2015; Iatsenko et al., 2013; Widjaja et al., 2015).

The computation of the CRC strength (CRCS), namely the degree of association between HP and respiration in the time direction from respiration to HP, is of paramount importance because it decreases with age (Iatsenko et al., 2013; Porta et al., 2014) and this decline provides information complementary to that derived from different autonomic control markers such as RSA (Laitinen et al., 2004; Beckers et al., 2006), cardiac baroreflex sensitivity (Laitinen et al., 1998; Milan-Mattos et al., 2018), cardiac control complexity (Kaplan et al., 1991; Catai et al., 2014) and the gain of the relation from respiration to HP (Saul et al., 1991). The relevance of assessing CRCS is further outlined by the well-known finding that it declines in situations evoking a high sympathetic tone and/or modulation such as during postural challenges (Porta et al., 2012, 2015) and it is altered in pathological conditions (Garcia et al., 2013; Schulz et al., 2013, 2015; Riedl et al., 2014; Penzel et al., 2016). Improving CRCS might be advisable because it would lead to a greater fraction of HPV driven by respiration and, as such, a more powerful and efficient cardiac vagal control might be in place irrespective of the efficiency of the cardiac baroreflex.

Physical exercise might improve CRC. Indeed, a moderate exercise training produces vagal control enhancement (Al-Ani et al., 1996) and it is exploited as a countermeasure to limit the decrease of the HPV magnitude with age (Albinet et al., 2010). Among the possible exercises the inspiratory muscle training (IMT) might be effective in improving CRCS. This position, taken in this study as a working hypothesis, is supported by numerous studies that suggested that IMT is able to improve RSA in both healthy and pathological subjects (Ferreira et al., 2013; Kaminski et al., 2015; Da Luz Goulart et al., 2016; Martins de Abreu et al., 2017; Karsten et al., 2018; Rodrigues et al., 2018). Even though the increase of RSA does not necessary imply an increase of CRCS (Eckberg and Karemaker, 2009; Porta et al., 2012, 2015), the unmodified cardiac baroreflex sensitivity observed after IMT of moderate intensity (DeLucia et al., 2018) prompts for a possible role of an empowered CRC to explain the after training elevation of RSA.

The aim of the study is to assess the effect of IMT training on CRCS in amateur cyclists. This population was chosen because these non-professional sportsmen should have a high vagal basal tone and CRCS that might be improved further with some difficulty, thus stressing more evidently the potential of IMT. The CRCS was measured using a more traditional non-causal technique such as the squared coherence (Saul et al., 1991) and more original causal methods based on the computation of transfer entropy (TE) (Barnett et al., 2009; Porta and Faes, 2016). Both a causal bivariate (Porta et al., 2018) and a causal trivariate (Porta et al., 2015) approach assessing the interactions from respiration to HPV unconditioned and conditioned on systolic arterial pressure (SAP) variability are exploited. This comparison is carried out to better understand the need of accounting for the influence of SAP variability when estimating CRCS to eventually discard the effects of respiration on HPV that are mediated by SAP changes at the respiratory frequency (RF) via the activation of cardiac baroreflex (Baselli et al., 1994; Nollo et al., 2005). CRCS is measured before (PRE) and after (POST) 11 weeks of IMT at rest in supine condition (REST) and during sympathetic activation induced by acting standing (STAND). Baroreflex control is monitored via sequence analysis (Bertinieri et al., 1985; Parati et al., 1988) to better understand its role in explaining the observed findings. Analysis was carried in three groups of amateur cyclists undergoing different IMT intensities.

## Materials and Methods

### Characterization of the Population

The full description of the population, justification of the sample size, description of the fitness state, characterization of the IMT and experimental protocol was reported in Martins de Abreu et al. (2019). Briefly, a total of 100 recreational male cyclists were screened for eligibility. Subjects were apparently healthy with age ranging from 20 to 40 years. They practiced cycling for 150 min per week, for at least 6 months. We excluded cyclists with alterations of the cardiac electric and/or respiratory activity as detectable during incremental treadmill exercise and cardiopulmonary tests, obese with body mass index larger than 30 kg⋅m^–2^, subjects with cardiovascular risk factors, smokers or former smokers with less than 1 year of interruption, habitual drinkers, drug abusers or recreational drug users, subjects who used drugs or medicines that could interfere with cardiac control and autonomic function, and who performed any type of IMT during the last 12 months. For the eligibility and the characterization of the population, cyclists underwent traditional anamnesis, conventional 12-lead electrocardiogram (ECG) at rest, treadmill exercise test, cardiopulmonary test for the assessment of peak oxygen uptake (peak VO_2_), and the evaluation of maximal inspiratory pressure (MIP) and maximal expiratory pressure (MEP).

The training protocol was registered in the ClinicalTrials.gov (NCT02984189) and the study was approved by the Human Research Ethics Committee of the Federal University of São Carlos (UFSCar) (Protocol: 1.558.731). The study adhered to the principles of the Declaration of Helsinki for research studies involving humans. All participants provided a written informed consent to participate in the study.

### IMT Protocol

Only 50 individuals met the eligibility criteria and were randomized into the three groups undergoing different intensities of IMT. Randomization process was based on the creation of groups formed by three subjects (i.e. triplets) with similar age and aerobic functional classification. Random allocation, performed via brown envelops, was carried out over these triplets. One smaller group of two individuals was created because 50 was not a multiple of 3. The three groups were composed by 17, 17 and 16 subjects. The first group performed a sham IMT (SHAM) of very low intensity against an inspiratory resistance of 6 cmH_2_O. The second group followed an IMT of moderate intensity against a respiratory resistance set to 60% of MIP (MIP60). The third group was trained at the critical inspiratory pressure (CIP) as determined in Rehder-Santos et al. (2019) and corresponding to an optimized high intensity IMT ranging from 80% to 90% of MIP (CIP). One subject was moved from the CIP group to the MIP60 one to avoid the retreat of this individual during the first session of the training. Therefore, SHAM, MIP60 and CIP groups were formed by 17, 18, and 15 subjects respectively. Some of subjects were excluded mainly because they did not conclude the training, namely 8, 8 and 3 cyclists in the SHAM, MIP60 and CIP groups respectively. Therefore, 9, 10, and 12 subjects concluded the SHAM, MIP60 and CIP training and could undergo the POST session of recording. Unfortunately, in 1 subject belonging to the CIP group the signals were of poor quality, thus allowing the analysis of the recordings of 9, 10, and 11 subjects in the SHAM, MIP60 and CIP groups respectively. The SHAM, MIP60 and CIP groups were similar in terms of age, body mass index, peak VO_2_, MIP and MEP as tested via one-way analysis of variance, or Kruskal–Wallis one-way analysis of variance on ranks when appropriate, applied to continuous variables, or χ^2^ test applied to aerobic functional classification (Martins de Abreu et al., 2019).

The subjects performed IMT for about 1 h, 3 days per week, for 11 weeks, using a linear inspiratory loading device PowerBreathe (Ironman K5, HaB Ltd, United Kingdom). The protocol was composed of a warm-up phase lasting 5 min during which each participant performed a constant loading protocol at 50% of his training load, followed by 3 consecutive IMT sessions of 15 min. The second and the third IMT sessions were preceded by 1-min recovery. During training, the subjects were instructed to maintain the breathing rate at 12 acts per minute and this rate was reinforced by a verbal command of the physiotherapist. Volunteers who did not complete the 3 weekly IMT sessions or the 11 full weeks of IMT, or modified their physical activities, physical training or lifestyles, or started using any supplement or medication during IMT were excluded.

### Experimental Protocol and Data Acquisition

The overall duration of the study was 13 weeks. Evaluation of cardiovascular control markers were carried out during the first and thirteenth weeks, just before and after IMT being the PRE and POST conditions respectively. For cardiovascular control assessment we acquired the ECG (lead MC5) via a bioamplifier (BioAmp FE132, ADInstruments, Australia), non-invasive continuous finger arterial pressure (Finometer Pro, Finapres Medical Systems, Netherlands) and respiratory movement (RM) through a thoracic belt (Marazza, Monza, Italy). Signals were sampled at 1000 Hz (Power Lab 8/35, ADInstruments, Australia). Recording sessions were carried out at the Cardiovascular Physical Therapy Laboratory, Department of Physical Therapy, UFSCar, São Carlos, Brazil according to standardized criteria minimizing individual and environmental factors that might increase the variance of cardiovascular control markers (Milan-Mattos et al., 2018). Subjects were initially maintained at REST for 10 min to stabilize the cardiovascular variables. After this period, signals were recorded for 15 min at REST. Then, the subject was asked to change posture and signals were acquired for additional 15 min during STAND. STAND session followed always REST. Throughout the procedure, subjects were instructed to breathe spontaneously and were not allowed to talk.

### Extraction of Beat-to-Beat Variability Series

The HP was determined over the ECG as the temporal distance between two consecutive R-wave peaks. The *i*th SAP was detected as the maximum of arterial pressure signal within the *i*th HP. The RM signal was sampled at the first R-wave delimiting the onset of the *i*th HP. Delineations of the R-wave peak and arterial pressure maximum were carefully checked to avoid erroneous detections or missed beats. If isolated ectopic beats affected HP and SAP, these measures were linearly interpolated using the closest values unaffected by ectopies. Since we were interested in short-term cardiac control, analyses were carried out over sequences of 256 consecutive HP, SAP and RM values (Task Force, 1996). The sequences were selected in a random position within REST and STAND periods. The random position was decided according to an automatic routine randomly extracting the onset of the segment from a uniform distribution of integers ranging from the session onset to the session offset (minus 256). The operator has no possibility to intervene on the selection. The procedure avoided the selection of the first 3 min of STAND. We computed the mean and variance of HP and SAP series, labeled as μ_HP_, σ^2^_HP_, μ_SAP_ and σ^2^_SAP_ and expressed in ms, ms^2^, mmHg and mmHg^2^ respectively. With the exception of the means, all the other markers were computed over linearly detrended sequences.

### Squared Coherence Analysis

The degree of linear coupling between HP and RM series as a function of the frequency f was computed via squared coherence function *K*^2^_HP–__RM_(f). The *K*^2^_HP–__RM_(f) is defined as the ratio between the square HP-RM cross-spectrum modulus divided by the product of the HP and RM power spectra. *K*^2^_HP–__RM_(f) ranges from 0 to 1, where 0 indicates perfect uncorrelation between HP and RM at the frequency f, while 1 indicates full correlation. The cross-spectrum and power spectra were estimated according to a parametric approach based on the bivariate autoregressive model (Porta et al., 2000). The coefficients of the model were identified via a traditional least squares technique and the order was fixed at 10 (Porta et al., 2000). *K*^2^_HP–RM_(f) was sampled in correspondence of the weighted average of the central frequency of the RM spectral components in high frequency (HF, from 0.15 to 0.4 Hz) band. This frequency was taken as an estimate of the RF. The sampling of the *K*^2^_HP–RM_(f) at the RF was referred to as *K*^2^_HP–__RM_(RF) and it is dimensionless.

### Model-Based Conditional and Unconditional TE

The degree of association in the temporal direction from a cause signal to an effect one was computed via TE measuring the amount of information transferred from the cause to the effect (Schreiber, 2000). Defined the restricted universe of knowledge as the set formed by the effect and all the possible confounding factors, the TE computes the reduction of information carried by the target signal when the restricted universe of knowledge is completed by including the presumed cause to become the full universe of knowledge. The greater the TE, the higher the association from the cause to the effect, the larger the coupling strength from the cause to the effect.

In this specific application the presumed cause is RM, the effect is HP and the possible confounding factor is SAP. At difference with *K*^2^_HP–RM_(f) the TE has the inherent advantage to be an asymmetric function, namely the TE from RM to HP is different from the TE from HP to RM. This feature makes TE to be particularly attractive in quantifying CRCS whether the degree of association in the temporal direction from HP to RM is present and larger than that in the reverse temporal direction. Indeed, in this situation *K*^2^_HP–RM_(f) would be dominated by mechanisms operating in the temporal direction that have nothing to do with CRC. The TE from RM to HP was computed in two different full universes of knowledge Ω_2_ = {HP, RM} and Ω_3_ = {HP, RM, SAP} respectively. Assigned Ω_2_ and Ω_3_ we defined Ω_2_\RM = {HP} and Ω_3_\RM = {HP, SAP} as the two restricted universes of knowledge built from Ω_2_ and Ω_3_ after excluding the presumed cause RM. The TE from RM to HP in Ω_2_, termed TE_R__M__→__HP_, was computed as a half of the logarithm of the prediction error variance of HP in Ω_2_\RM to that of HP in Ω_2_ (Barnett et al., 2009; Porta et al., 2018). The TE from RM to HP in Ω_3_ conditioned on SAP, termed TE_R__M__→__HP| SAP_, was computed as a half of the logarithm of the prediction error variance of HP in Ω_3_\RM to that of HP in Ω_3_ (Barnett et al., 2009; Porta et al., 2015). Both TE_R__M__→__HP_ and TE_R__M__→__HP| SAP_ were dimensionless.

In this specific application a model-based approach based on multivariate linear regression models, namely the class of the autoregressive model with exogenous input (Baselli et al., 1997), was exploited to fit the series in Ω_2_, Ω_2_\RM, Ω_3_, and Ω_3_\RM. After normalizing HP, SAP, and RM series to have zero mean and unit variance by subtracting the mean and by dividing the result by the standard deviation, the coefficients of the models were identified via traditional least squares approach and Cholesky decomposition method (Baselli et al., 1997). In the computation of TE_RM__→__HP_ and TE_RM__→__HP| SAP_ the model order was optimized in the range from 8 to 16 according to the Akaike’s figure of merit (Akaike, 1974) computed in Ω_2_ and Ω_3_ respectively. The prediction error was computed as the difference between the current value of the HP series and its best prediction provided by the model. Immediate effects (i.e. within the current HP) from SAP and RM to HP were considered in agreement with the fastness of the vagal actions characterizing both cardiac baroreflex, namely the link from SAP to HP, and CRC, namely the pathway from RM to HP (Eckberg, 1976; Porta et al., 2013b). All the regressions have the same number of coefficients equal to the optimal model order. The models in Ω_2_\RM and Ω_3_\RM were separately identified using the optimal model order estimated in Ω_2_ and Ω_3_ respectively (Porta et al., 2015).

### Cardiac Baroreflex Evaluation

The sequence technique is one of the most utilized methods for the characterization of cardiac baroreflex from spontaneous HP and SAP variability series (Bertinieri et al., 1985; Parati et al., 1988). We applied the sequence technique as implemented in Porta et al. (2000, 2013a). More specifically, we defined as HP-SAP pattern of baroreflex origin an HP-SAP joint scheme featuring three consecutive and contemporaneous HP and SAP increases or decreases. Therefore, an HP-SAP pattern of baroreflex origin is characterized by same-sign HP and SAP ramps with a delay between them equal to 0 beats, thus focusing on the fast vagal arm of the cardiac baroreflex featuring very short latencies compatible with the measurement convention adopted in this study (Eckberg, 1976; Milan-Mattos et al., 2018). All the detected HP-SAP patterns of baroreflex origin were retained in this analysis regardless of the magnitude of total, or partial, SAP and HP variations and the strength of the linear association between HP and SAP values (Porta et al., 2013a). The baroreflex sensitivity (BRS) was computed as the mean of the slopes of the regression lines of HP on SAP calculated over all HP-SAP patterns of baroreflex origin. BRS was positive by definition and expressed in ms⋅mmHg^–1^. The percentage of the HP-SAP patterns of baroreflex origin with respect to the overall amount of HP-SAP joint schemes (SEQ%) was assessed as well and taken as a measure of the degree of involvement of cardiac baroreflex control. By definition, SEQ% ranged between 0 and 100.

### Statistical Analysis

Normality was tested via Shapiro–Wilk test. The assessment of the effect of IMT on time domain, cardiac baroreflex and CRCS indexes was carried out within an assigned group of athletes via two-way repeated measures analysis of variance (Holm–Sidak correction for multiple comparisons). The significance of the effect of training within the same experimental condition (i.e. REST or STAND) and the response to postural challenge within the same period of analysis (i.e. PRE or POST) was tested. Assigned the group of subject, if the null hypothesis of normal distribution of a given variable was rejected in some experimental condition or period of analysis, the values of that variable in all experimental conditions and periods of analysis were log-transformed before performing two-way repeated measures analysis of variance. No formal statistical analysis was carried out among different groups (i.e. SHAM, MIP60 and CIP). Comparison among different groups was qualitative and based on the observation of significances detected by the previously mentioned two-way repeated measures approach. Data are expressed as mean ± standard deviation. Statistical analysis was carried out using a commercial statistical program (Sigmaplot, v.14.0, Systat Software, Inc., Chicago, IL, United States). A *p* < 0.05 was always considered statistically significant.

## Results

Time domain markers are summarized in Table 1. The effect of STAND was significant over μ_HP_ regardless of the training status (i.e. PRE and POST) and type of training (i.e. SHAM, MIP60, and CIP). SHAM and MIP60 trainings lengthened μ_HP_ at REST respectively, while CIP training shortened μ_HP_ during STAND. σ^2^_HP_ and μ_SAP_ were not influenced by experimental condition and training status. This finding held irrespective of the type of training. σ^2^_SAP_ did not vary with the training status but it was affected by the postural challenge. Indeed, in both PRE and POST sessions σ^2^_SAP_ increased during STAND and this result held only in the MIP60 group. In the SHAM and CIP groups RF was not affected by either experimental condition or training status. A post-training decrease of RF was observed in the MIP60 group at REST, while no effect of training was visible during STAND. In the same group orthostatic challenge did not influence the RF regardless of the training status.

**TABLE 1:** Time domain HP and SAP markers and RF during SHAM, MIP60, and CIP trainings.

Index	Experimental condition	SHAM		MIP60		CIP	
				
		REST	STAND	REST	STAND	REST	STAND
μ_HP_ [ms]	PRE	990113	810135*	992181	815165*	94676	79171*
	POST	1063133§	876178*	1106175§	84998*	928109	73795§*
σ^2^_HP_ [ms^2^]	PRE	42893631	33921774	26071850	35823978	28592745	23691508
	POST	51903647	46953980	41492658	40003821	18561390	16501388
μ_SAP_ [mmHg]	PRE	11117	10416	11214	11618	1109	10914
	POST	9838	9539	11223	11424	11316	11516
σ^2^_SAP_ [mmHg^2^]	PRE	3222	5220	155	4120*	2313	2912
	POST	3625	3830	2313	4629*	3016	3925
RF [apm]	PRE	15.93.7	14.03.4	18.34.5	15.84.9	17.03.7	15.73.2
	POST	16.41.8	15.93.9	15.25.0§	16.45.3	18.94.2	17.93.8

IMT, inspiratory muscle training; SHAM, sham IMT; MIP60, IMT at 60% of the maximum inspiratory pressure; CIP, IMT training at the critical inspiratory pressure; REST, at rest in supine position; STAND, active standing; HP, heart period; SAP, systolic arterial pressure; μ_*HP*_, HP mean; σ^2^_*HP*_, HP variance; μ_*SAP*_, SAP mean; σ^2^_*SAP*_, SAP variance; RF, respiratory frequency expressed in acts per minute (apm). Data are presented as mean ± standard deviation. The symbol * indicates *p* < 0.05 vs. REST within the same period of analysis (i.e. PRE or POST) assigned the training group. The symbol § indicates *p* < 0.05 versus POST within the same experimental condition (i.e. REST or STAND) assigned the training group.

**FIGURE 1:**
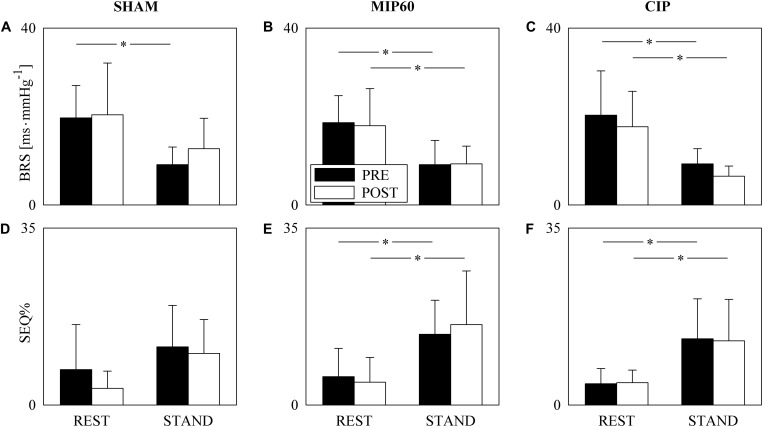
The grouped vertical bar graphs show BRS **(A–C)** and SEQ% **(D–F)** before (PRE, black bars) and after (POST, white bars) training as a function of the experimental condition (i.e. REST and STAND) in the three considered groups, namely SHAM **(A,D)**, MIP60 **(B,E)**, and CIP **(C,F)**. The values are reported as mean plus standard deviation. The symbol ^∗^ indicates a statistically significant difference versus REST within the same training condition (i.e. PRE or POST) with *p* < 0.05.

The grouped vertical bar graphs of Figure 1 show BRS and SEQ% computed over SHAM (Figures 1A,D), MIP60 (Figures 1B,E), and CIP (Figures 1C,F) groups in PRE (black bars) and POST (white bars) sessions as a function of the experimental condition (i.e. REST and STAND). STAND decreased BRS and increased SEQ%. This tendency held regardless of the training condition (i.e. PRE and POST) and was observed in all the groups (i.e. SHAM, MIP60 and CIP). However, the effect of STAND over BRS and SEQ% was more powerful in MIP60 (Figures 1B,E) and CIP (Figures 1C,F) groups than in the SHAM one (Figures 1A,D). IMT did not influence BRS and SEQ% given that no significant PRE-POST difference was observed either at REST or during STAND irrespective of the IMT intensity.

**FIGURE 2:**
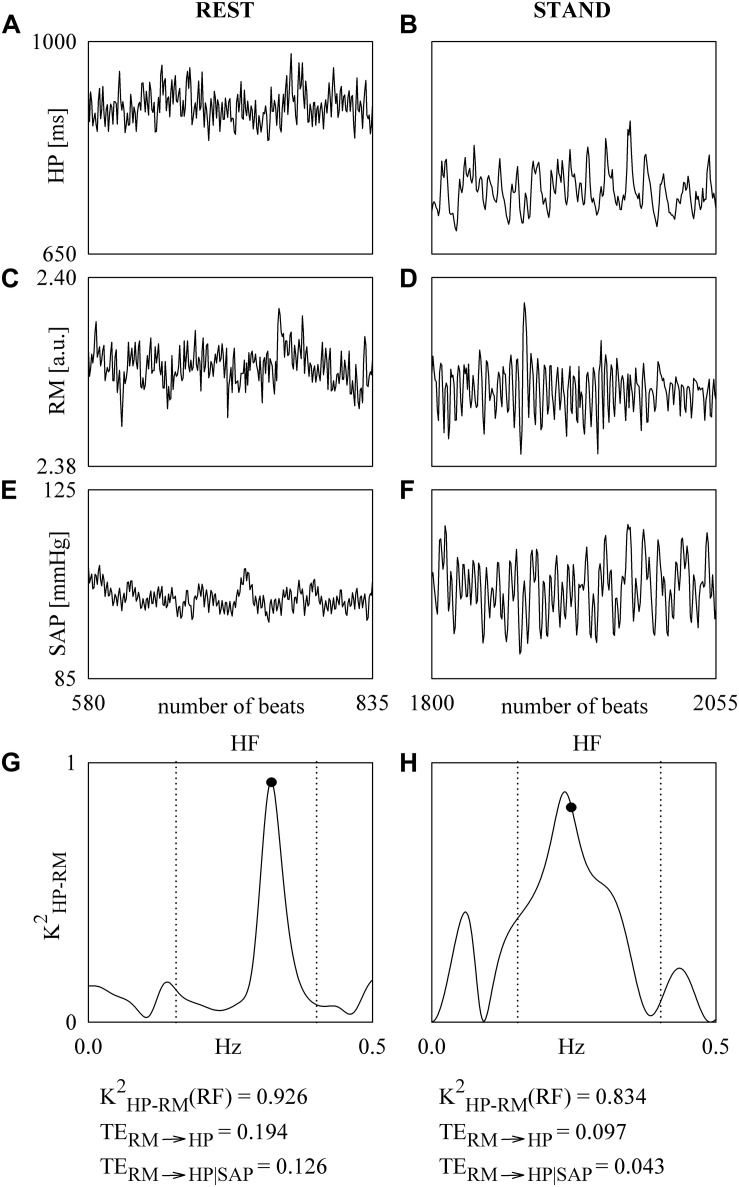
The line plots show the series of HP **(A,B)**, RM **(C,D)**, and SAP **(E,F)**. The series are recorded from a SHAM subject at REST **(A,C,E)** and during STAND **(B,D,F)**. The *K*^2^_HP–SAP_ functions computed over the HP and RM series at REST and during STAND are shown in **(G,H)**. The sampling of the *K*^2^_HP–SAP_ function at the RF is indicated by a solid circle in **(G,H)**. The limits of the HF band are given as dotted lines in **(G,H)**. The values of the *K*^2^_HP–__RM_(RF), TE_RM__→__HP_, and TE_RM__→__HP| SAP_ are reported below the panels representing *K*^2^_HP–RM_.

Figure 2 shows an example of the *K*^2^_HP–RM_ and TE analyses performed over series recorded at REST and during STAND in a subject belonging to the SHAM group. The series of HP, RM and SAP acquired at REST and during STAND are shown in Figures 2A,C,E and Figures 2B,D,F respectively. During STAND μ_HP_ decreases and σ^2^_SAP_ increases. The corresponding K^2^_HP–RM_ functions are reported in Figures 2G,H respectively with the indication of the inferior and superior limit of the HF band (dotted lines) and sampling at the RF (solid circle). The values of the *K*^2^_HP–__RM_(RF), TE_RM__→__HP_, and TE_RM__→__HP| SAP_ are given below the panels representing *K*^2^_HP–RM_. The values of *K*^2^_HP–__RM_(RF), TE_RM__→__HP_, and TE_RM__→__HP| SAP_ at REST are higher than the correspondent value during STAND, thus indicating a reduced cardiorespiratory coupling with the postural challenge. TE_RM__→__HP_ is larger than TE_RM__→__HP| SAP_, thus suggesting that a portion of the information transferred from RM to HP is mediated by SAP changes.

**FIGURE 3:**
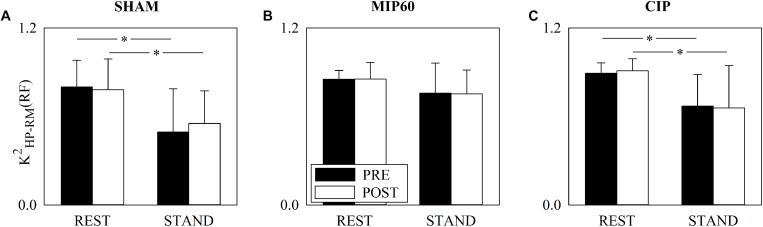
The grouped vertical bar graphs show *K*^2^_HP–__RM_(RF) before (PRE, black bars) and after (POST, white bars) training as a function of the experimental condition (i.e. REST and STAND) in the three considered groups, namely SHAM **(A)**, MIP60 **(B)**, and CIP **(C)**. The values are reported as mean plus standard deviation. The symbol ^∗^ indicates a statistically significant difference versus REST within the same training condition (i.e. PRE or POST) with *p* < 0.05.

The grouped vertical bar graphs of Figure 3 show *K*^2^_HP–__RM_(RF) computed over SHAM (Figure 3A), MIP60 (Figure 3B), and CIP (Figure 3C) groups in PRE (black bars) and POST (white bars) sessions as a function of the experimental condition (i.e. REST and STAND). All the groups responded to the orthostatic challenge by decreasing *K*^2^_HP–__RM_(RF) in both PRE and POST sessions. However, the decrease was significant solely in SHAM (Figure 3A) and CIP (Figure 3C) groups, while in the MIP60 group the reduction was not significant (Figure 3B). Regardless of the IMT intensity no effect of training was visible over *K*^2^_HP–__RM_(RF) both at REST and during STAND.

**FIGURE 4:**
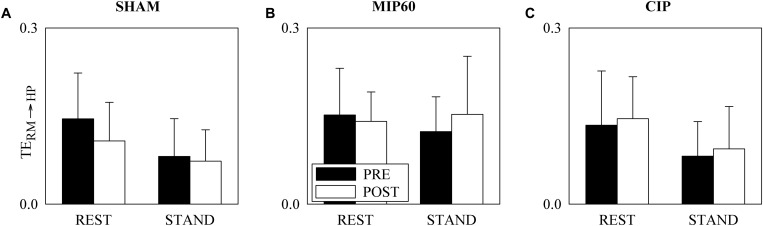
The grouped vertical bar graphs show TE_RM__→__HP_ before (PRE, black bars) and after (POST, white bars) training as a function of the experimental condition (i.e. REST and STAND) in the three considered groups, namely SHAM **(A)**, MIP60 **(B)**, and CIP **(C)**. The values are reported as mean plus standard deviation.

Figure 4 has the same structure as Figure 3 but it shows TE_RM__→__HP_. This parameter did not change with either training status (i.e. PRE and POST) or experimental condition (i.e. REST and STAND). This conclusion held regardless of the type of training, namely SHAM (Figure 4A), MIP60 (Figure 4B), and CIP (Figure 4C).

**FIGURE 5:**
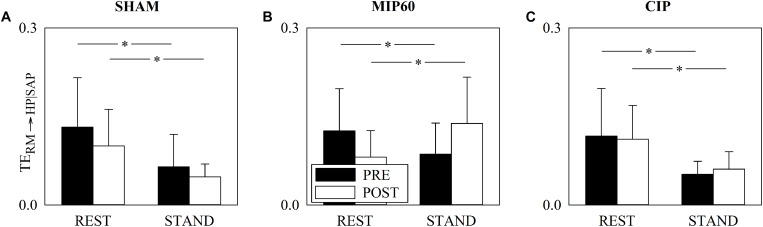
The grouped vertical bar graphs show TE_RM__→__HP| SAP_ before (PRE, black bars) and after (POST, white bars) training as a function of the experimental condition (i.e. REST and STAND) in the three considered groups, namely SHAM **(A)**, MIP60 **(B)**, and CIP **(C)**. The values are reported as mean plus standard deviation. The symbol ^∗^ indicates a statistically significant difference versus REST within the same training condition (i.e. PRE or POST) with *p* < 0.05.

Figure 5 has the same structure as Figure 3 but it shows TE_RM__→__HP| SAP_. Regardless of the training condition (i.e. PRE or POST) STAND influenced TE_RM__→__HP| SAP_. This consideration held for all the groups. Remarkably, the sign of the TE_RM__→__HP| SAP_ variation induced by STAND depended on the intensity of the IMT training. Indeed, in POST condition, while STAND decreased TE_RM__→__HP| SAP_ in SHAM (Figure 5A) and CIP (Figure 5C) group, the postural challenge significantly increased TE_RM__→__HP| SAP_ in the MIP60 group (Figure 5B). At REST the decrease of TE_RM__→__HP| SAP_ in response to STAND was significant regardless of the group (Figures 5A–C). No PRE-POST difference was detected both at REST and during STAND (Figures 5A–C) and this result held for all the groups.

## Discussion

The main finding of this study can be summarized as follows: (i) in PRE condition sympathetic activation and vagal withdrawal induced by postural challenge reduced CRCS even though the significance of the decrease depends on the CRC marker; (ii) in MIP60 group a causal CRCS marker conditioning SAP out can detect the post-training increase of CRCS during STAND, while more traditional non-causal and simpler causal CRCS indexes cannot; (iii) in MIP60 group the post-training increase of TE_RM__→__HP| SAP_ induced by STAND was observed without significant modifications of μ_SAP_ and RF and in presence of the expected response of μ_HP_ and BRS to STAND; (iv) CIP was not able to prevent the decrease of CRCS in response to STAND.

### On the Need of a Causal Approach Conditioning SAP Variability Out for the Evaluation of CRCS

The quantification of CRCS necessitates the computation of the association between RM and HP dynamics in a specific time direction (i.e. from RM to HP) and the possibility of conditioning out any signal that might act as a confounding factor masking or biasing the considered HP-RM association. Signal processing tools assessing causality are suitable candidates for the evaluation of CRCS because the temporal direction of the dynamical interactions can be accounted for, confounding factors can be easily conditioned out, and the computed metrics might have attracting features such as being dimensionless and bounded (e.g. TE is bounded between 0 and the Shannon entropy of HP series) (Porta and Faes, 2016). In this study we exploited a model-based approach assessing the information transferred from RM to HPV as the reduction of information carried by the HP series resulting from the acquisition of the RM signal in addition to HP and SAP variability series (Barnett et al., 2009; Porta et al., 2015). It can be argued that the possibility of imposing a temporal direction of interactions (i.e. from RM to HP) might be irrelevant in assessing CRC because it is unlikely that modifications of HP could affect respiratory centers because no anatomical feedback from HP to RM does exist. However, given that the hypothesis of open loop relation from RM to HP has been repeatedly rejected in experiment conditions commonly exploited in cardiac autonomic control analysis and with respiratory signals routinely acquired in many laboratories (Yana et al., 1993; Porta et al., 2013b), some caution about the use of non-causal tools such as *K*^2^_HP–RM_(f) is advisable. An active pathway on the reverse time direction (i.e. from HP to RM) might be the mere consequence of the different rapidity of the recorded variables to respond to respiratory center inputs (Yana et al., 1993; Porta et al., 2013b). The possibility of conditioning out confounding factors is even more important than that of imposing causality. Indeed, it is well-known that the association between RM and HPV might be mediated by cardiac baroreflex (Porta et al., 2012) solicited by SAP fluctuations resulting from modifications of the venous return driven by respiratory-related changes of intrathoracic pressure (Toska and Eriksen, 1993; Caiani et al., 2000). Accounting for baroreflex influences on the HP-RM link might be important in our experimental protocol given that IMT generates remarkable modifications of intrathoracic pressures (Lurie et al., 2002; Convertino et al., 2004b; Vranish and Bailey, 2015), that might have some impact on baroreflex responses (Angell James, 1971), and given that sympathetic activation evoked by STAND is mediated by the baroreflex engagement (Taylor and Eckberg, 1996; Porta et al., 2011, 2012). If these influences were not accounted for, the association between RM and HP dynamics might be biased by the simultaneous action of pathways other than the directed action of RM on HRV. Therefore, it is not surprising to find out that the statistical power of the TE_RM__→__HP| SAP_ is greater than that of a rougher causal marker that does not take into account SAP, such as the TE_RM__→__HP_. Even the modifications of *K*^2^_HP–__RM_(RF) with STAND might be the sole consequence of disregarding SAP dynamics. Indeed, the decrease of *K*^2^_HP–__RM_(RF) during STAND might be the genuine result of the reduction of the cardiac baroreflex sensitivity at the RF in response to the postural stimulus (Cooke et al., 1999; De Maria et al., 2019) limiting the strength of the HP-RM link mediated by SAP changes.

### Effect of the Orthostatic Challenge on CRCS in PRE Condition

This study confirms that orthostatic challenge determines a reduction of CRCS. Indeed, in PRE condition all the considered CRCS markers reached the same conclusion, even though with different statistical power. Indeed, the reduction of TE_RM__→__HP_ during STAND was less evident than that of *K*^2^_HP–__RM_(RF) and TE_RM__→__HP| SAP_. A CRCS decrease, proportional to the magnitude of the postural stimulus (i.e. the tilt table inclination during graded head-up tilt test), was observed via a causal method decomposing the variance of HP series using a multivariate partial power spectral decomposition technique (Porta et al., 2012) and via a model-based conditional TE approach (Porta et al., 2015). The sympathetic activation and vagal withdrawal associated to the orthostatic challenge (Montano et al., 1994; Cooke et al., 1999; Furlan et al., 2000; Marchi A. et al., 2016) is likely to be responsible for the decoupling of the respiratory rhythm modulating vagal drive and HP dynamics. This decoupling is favored by the decrease of the gain of the HP-RM transfer function (Saul et al., 1991; Yana et al., 1993) and by the reduction of the RSA (Pomeranz et al., 1985) known to occur when cardiac vagal control is limited like during parasympathetic blockade performed via high dose administration of atropine. The reduction of the CRCS is robustly detected in presence of a shift of the sympathovagal balance toward a sympathetic predominance even when this unbalance is not evoked by an orthostatic challenge such as during healthy aging (Iatsenko et al., 2013; Nemati et al., 2013; Porta et al., 2014), thus stressing the inverse relationship between markers of CRC and vagal control and the possibility to use CRCS as a further marker of vagal responsiveness of the sinus node.

### Effect of the Orthostatic Challenge on CRCS in POST Condition

The effects of IMT on CRCS seem to be of limited entity both at REST and during STAND. Indeed, no significant difference between PRE and POST was observed and this finding held irrespective of the IMT intensity and type of CRCS marker. However, some influences of IMT became visible when the response to STAND was analyzed in POST condition. Indeed, TE_RM__→__HP| SAP_ decreased during STAND compared to REST in both SHAM and CIP groups, while it increased in the MIP60 group. The post-training increase of CRCS induced by STAND was not detectable via *K*^2^_HP–__RM_(RF) and TE_RM__→__HP_ likely due to the limited ability of a non-causal index of CRC, such as *K*^2^_HP–__RM_(RF), and of a simpler causal marker that does not account for confounding factors such as TE_RM__→__HP_. It seems that after MIP60 training amateur cyclists could cope with the postural stressor with an increased CRC in presence of a usual response of sympathetic and vagal sympathetic branches of the autonomic nervous system to the postural stressor as denoted by the decrease of μ_HP_ and increase of σ^2^_SAP_. The mechanism underlying the improvement of CRCS during STAND induced by the MIP60 training is unclear. Breathing through an inspiratory resistance of limited value decreases intrathoracic pressure and increases stroke volume, cardiac output and SAP (Lurie et al., 2002; Convertino et al., 2004b). Given the rhythmical nature of respiration, modifications of intrathoracic pressure and, consequently, of stroke volume (Toska and Eriksen, 1993; Caiani et al., 2000), periodically solicit the stretch-sensitive areas of barosensory vessels located in thorax (Angell James, 1971) and this dynamical stimulation might be responsible for producing post-training beneficial effects in terms of augmented vessel elasticity and reduced mechanical stiffness. This mechanism was advocated to explain the improved baroreflex control after IMT of light-to-moderate intensity in patients suffering for orthostatic hypotension as a consequence of spinal cord injury (Aslan et al., 2016). However, this baroreflex-mediated mechanism cannot explain the complexity of our results and findings present in literature. Indeed, if an IMT of moderate intensity was able to empower baroreflex, we would expect a greater after-training BRS. Conversely, no PRE-POST variation of BRS was observed and the usual trend of baroreflex markers with STAND was detected after MIP60 training. The lack of influences on the baroreflex regulation is in agreement with data derived during a session of breathing through an inspiratory resistance of small value (Convertino et al., 2004a) and with the long-term effects of an IMT training of moderate intensity (DeLucia et al., 2018). We suggest that the post-training changes of CRCS detected by TE_RM__→__HP| SAP_ during STAND in MIP60 group are due to mechanisms unrelated to baroreflex. Also modifications of RF cannot explain this finding given that RF did not change in MIP60 group during STAND. We speculate that the MIP60 training might have promoted central respiratory network modifications (Spyer, 1995; Eckberg, 2003) through the solicitation of the afferent pulmonary and atrial stretch-activated neural circuits during training (Seals et al., 1990; Taha et al., 1995; Eckberg and Karemaker, 2009; Crystal and Salem, 2012). Remarkably, the post-training increase of CRCS was detected only by TE_RM__→__HP| SAP_ likely because the unvaried action of baroreflex might act as a confounding factor for the direct relation from RM to HP. Moreover, since this effect of the MIP60 training was visible solely during the sympathetic activation induced by the orthostatic stimulus, we speculate that this IMT might not produce exclusively modifications of the interactions between respiratory centers and vagal activity but also with central sympathetic drive. Remarkably, the post-training increase of CRCS during STAND observed in the MIP60 group was not detected in SHAM and CIP groups likely because the intensity of the SHAM training was too low to produce any post-training modifications of the cardiac control, while the CIP training might be ineffective. The ineffectiveness of CIP training might be related to the inability, compared to MIP60, to produce really important transmural pressures, namely the difference between intramural atrial pressure and extramural intrathoracic pressure being the net stimulus for the low pressure receptors in the atria. We conjecture that this inability prevents the generation of empowered variations of the afferent neural activity during the CIP training and, consequently, the possibility to stimulate some rearrangements at the level of central respiratory network. This supposition needs to be corroborated by the observation of additional variables during CIP session such venous return, atrial and intrathoracic pressure and stroke volume.

### Limitations of the Study and Future Developments

We remark the exploratory value of this study and recommend taking conclusions as hypotheses that should be tested over groups of larger size. With the results of the present work in mind future studies should be focused on a single type of IMT to concentrate the experimental effort on a single group of individuals. It is worth noting that at REST the MIP60 training was able to evoke a significant bradycardia in presence of an unvaried SAP. This finding is in contrast with some studies suggesting that IMT of moderate intensity could lower arterial pressure while leaving unmodified HP values in both normotensive (Vranish and Bailey, 2015; DeLucia et al., 2018) and hypertensive (Ferreira et al., 2013) subjects. Even the improvement of arterial pressure regulation, reported in subjects with orthostatic hypotension such as those after spinal cord injury (Aslan et al., 2016), it is not evident in our study given that the baroreflex control is not affected by IMT. These considerations suggest that, although belonging to the class of IMT, our modality of IMT might lead to long-term effects different from those reported in literature and/or long-term consequences depending on the trained population, thus stressing the need of standardization of the IMT to better control its chronic effects according to the type of treated subjects. This standardization appears to be mandatory especially whether IMT of moderate intensity is to be proposed as a standard practice in physiotherapy and sports medicine.

## Conclusion

This study suggests that an IMT of moderate intensity, such as MIP60, improves cardiac autonomic control by acting on CRC and this improvement is visible using a causal tool conditioning SAP out and only under a sympathetic stressor such as STAND. This improvement appears to be independent of cardiac baroreflex because the usual trends of BRS with STAND are detected after the MIP60 training. The present result indicates that the long-term effects of IMT of moderate intensity might be not limited to lower arterial pressure and vascular resistance (DeLucia et al., 2018) but they might be even wider through the possible involvement of regulatory centers in the brain stem that are not specifically devoted to arterial pressure control. This improvement is associated exclusively with an IMT training of moderate intensity. We suggest that the favorable effects of IMT of moderate intensity observed in patients featuring a high sympathetic drive (Ferreira et al., 2013; Da Luz Goulart et al., 2016; Martins de Abreu et al., 2017) and in healthy old subjects (Rodrigues et al., 2018) could be related to an improved CRC more evident under sympathetic stressor. The exploration of the mechanisms underlying the effects of MIP60 training might favor its specific application as a countermeasure of the progressive increase of sympathetic drive contributing to the decrease of CRCS in physiological and pathological situations.

## Data Availability Statement

The datasets generated for this study are available on request to the corresponding author.

## Ethics Statement

The studies involving human participants were reviewed and approved by the Human Research Ethics Committee of the Federal University of São Carlos (UFSCar) under the protocol 1.558.731. The patients/participants provided their written informed consent to participate in this study.

## Author Contributions

AP conceived and designed the study. RA, PR-S, CDS, ÉS, and CAS performed the experiments. RA and BC analyzed the data. RA and AP drafted the manuscript and prepared the figures. RA, AC, BC, PR-S, CDS, ÉS, CAS, and AP interpreted the results, edited and revised the manuscript, and approved the final version of the manuscript.

## Conflict of Interest

The authors declare that the research was conducted in the absence of any commercial or financial relationships that could be construed as a potential conflict of interest. The reviewer MJ declared a past collaboration with one of the authors AP to the handling Editor.

## Abbreviations

μ: mean
σ^2^: variance
BRS: baroreflex sensitivity
CIP: IMT at the critical inspiratory pressure
CRC: cardiorespiratory coupling
CRCS: CRC strength
ECG: electrocardiogram
HP: heart period
HPV: HP variability
IMT: inspiratory muscle training
*K*^2^: squared coherence function
MEP: maximal expiratory pressure
MIP: maximal inspiratory pressure
MIP60: IMT against a respiratory resistance set to 60% of MIP
POST: after 11 weeks of IMT
PRE: before 11 weeks of IMT
REST: at rest in supine position
RF: respiratory frequency
RM: respiratory movement signal
RSA: respiratory sinus arrhythmia
SAP: systolic arterial pressure
SEQ%: percentage of HP-SAP pattern of baroreflex origin
SHAM: IMT against an inspiratory resistance of 6 cmH_2_O
STAND: active standing
TE: transfer entropy
VO_2_: oxygen uptake.

## References

[B1] Akaike H. (1974). A new look at the statistical novel identification. *IEEE Trans. Autom. Control* 19 716–723. 10.1109/tac.1974.1100705

[B2] Al-Ani M. Munir S. M. White M. Townend J. Coote J. H. (1996). Changes in R-R variability before and after endurance training measured by power spectral analysis and by the effect of isometric muscle contraction. *Eur. J. Appl. Physiol.* 74 397–403. 10.1007/bf02337719 8954286

[B3] Albinet C. T. Boucard G. Bouquet C. A. Audiffren M. (2010). Increased heart rate variability and executive performance after aerobic training in the elderly. *Eur. J. Appl. Physiol.* 109 617–624. 10.1007/s00421-010-1393-y 20186426

[B4] Angell James J. E. (1971). The effects of changes of extramural, intrathoracic, pressure on aortic arch baroreceptors. *J. Physiol.* 214 89–103. 10.1113/jphysiol.1971.sp009420 5575380 PMC1331823

[B5] Aslan S. C. Randall D. C. Krassioukov A. V. Phillips A. Ovechkin A. V. (2016). Respiratory training improves blood pressure regulation in individuals with chronic spinal cord injury. *Arch. Phys. Med. Rehabil.* 97 964–973. 10.1016/j.apmr.2015.11.018 26718236 PMC4884550

[B6] Barnett L. Barrett A. B. Seth A. K. (2009). Granger causality and transfer entropy are equivalent for Gaussian variables. *Phys. Rev. Lett.* 103:238701. 20366183 10.1103/PhysRevLett.103.238701

[B7] Bartsch R. Kartelhardt J. W. Penzel T. Havlin S. (2007). Experimental evidence for phase synchronization transitions in the human cardiorespiratory system. *Phys. Rev. Lett.* 98:054102. 17358862 10.1103/PhysRevLett.98.054102

[B8] Baselli G. Cerutti S. Badilini F. Biancardi L. Porta A. Pagani M. (1994). Model for the assessment of heart period and arterial pressure variability interactions and of respiration influences. *Med. Biol. Eng. Comput.* 32 143–152. 10.1007/bf02518911 8022210

[B9] Baselli G. Porta A. Rimoldi O. Pagani M. Cerutti S. (1997). Spectral decomposition in multichannel recordings based on multivariate parametric identification. *IEEE Trans. Biomed. Eng.* 44 1092–1101. 10.1109/10.641336 9353988

[B10] Beckers F. Verheyden B. Aubert A. E. (2006). Aging and nonlinear heart rate control in a healthy population. *Am. J. Physiol.* 290 H2560–H2570. 16373585 10.1152/ajpheart.00903.2005

[B11] Bertinieri G. di Rienzo M. Cavallazzi A. Ferrari A. U. Pedotti A. Mancia G. (1985). A new approach to analysis of the arterial baroreflex. *J. Hypertens Suppl.* 3 S79–S81. 2856787

[B12] Bracic Lotric M. Stefanovska A. (2000). Synchronization and modulation in the human cardiorespiratory system. *Physica A* 283 451–461. 10.1016/s0378-4371(00)00204-1

[B13] Caiani E. G. Turiel M. Muzzupappa S. Porta A. Baselli G. Pagani M. (2000). Evaluation of respiratory influences on left ventricular function parameters extracted from echocardiographic acoustic quantification. *Physiol. Meas.* 21 175–186. 10.1088/0967-3334/21/1/321 10720013

[B14] Catai A. M. Takahashi A. C. M. Perseguini N. M. Milan J. Minatel V. Rehder-Santos P. (2014). Effect of the postural challenge on the dependence of the cardiovascular control complexity on age. *Entropy* 16 6686–6704. 10.3390/e16126686

[B15] Convertino V. A. Ratliff D. A. Ryan K. L. Cooke W. H. Doerr D. F. Ludwig D. A. (2004a). Effects of inspiratory impedance on the carotid-cardiac baroreflex response in humans. *Clin. Auton. Res.* 14 240–248. 15316841 10.1007/s10286-004-0180-4

[B16] Convertino V. A. Ratliff D. A. Ryan K. L. Doerr D. F. Ludwid D. A. Muniz G. W. (2004b). Hemodynamics associated with breathing through an inspiratory impedance threshold device in human volunteers. *Crit. Care Med.* 32 S381–S386. 15508665 10.1097/01.ccm.0000134348.69165.15

[B17] Cooke W. H. Hoag J. B. Crossman A. A. Kuusela T. A. Tahvanainen K. U. O. Eckberg D. L. (1999). Human responses to upright tilt: a window on central autonomic integration. *J. Physiol.* 517 617–628. 10.1111/j.1469-7793.1999.0617t.x 10332107 PMC2269357

[B18] Crystal G. J. Salem M. R. (2012). The Bainbridge and the “reverse” Bainbridge reflexes: history, physiology, and clinical relevance. *Anesth. Analg.* 114 520–532. 10.1213/ANE.0b013e3182312e21 21965361

[B19] Da Luz Goulart C. Simon J. C. De Borba Schneiders P. San Martin E. A. Cabiddu R. Borghi-Silva A. (2016). Respiratory muscle strength effect on linear and nonlinear heart rate variability parameters in COPD patients. *Int. J. Chron. Obstruct. Pulmon. Dis.* 11 1671–1677. 10.2147/COPD.S108860 27555757 PMC4968685

[B20] De Maria B. Bari V. Cairo B. Vaini E. Esler M. Lambert E. (2019). Characterization of the asymmetry of the cardiac and sympathetic arms of the baroreflex from spontaneous variability during incremental head-up tilt. *Front. Physiol.* 10:342. 10.3389/fphys.2019.00342 31001137 PMC6454064

[B21] DeLucia C. M. De Asis R. M. Bailey E. F. (2018). Daily inspiratory muscle training lowers blood pressure and vascular resistance in healthy men and women. *Exp. Physiol.* 103 201–211. 10.1113/EP086641 29178489

[B22] Eckberg D. L. (1976). Temporal response patterns of the human sinus node to brief carotid baroreceptor stimuli. *J. Physiol.* 258 769–782. 10.1113/jphysiol.1976.sp011445 978502 PMC1309004

[B23] Eckberg D. L. (2003). The human respiratory gate. *J. Physiol.* 548 339–352. 10.1111/j.1469-7793.2003.00339.x 12626671 PMC2342859

[B24] Eckberg D. L. Karemaker J. M. (2009). Point:counterpoint: respiratory sinus arrhythmia is due to a central mechanism vs. respiratory sinus arrhythmia is due to the baroreflex mechanism. *J. Appl. Physiol.* 106 1740–1744. 18719228 10.1152/japplphysiol.91107.2008

[B25] Ferreira J. B. Plentz R. D. Stein C. Casali K. R. Arena R. Lago P. D. (2013). Inspiratory muscle training reduces blood pressure and sympathetic activity in hypertensive patients: a randomized controlled trial. *Int. J. Cardiol.* 166 61–67. 10.1016/j.ijcard.2011.09.069 21985749

[B26] Furlan R. Porta A. Costa F. Tank J. Baker L. Schiavi R. (2000). Oscillatory patterns in sympathetic neural discharge and cardiovascular variables during orthostatic stimulus. *Circulation* 101 886–892. 10.1161/01.cir.101.8.886 10694528

[B27] Garcia A. J. Koschnitzky J. E. Dashevskiy T. Ramirez J.-M. (2013). Cardiorespiratory coupling in health and disease. *Auton. Neurosci.* 175 26–37. 10.1016/j.autneu.2013.02.006 23497744 PMC3683976

[B28] Hirsch J. A. Bishop B. (1981). Respiratory sinus arrhythmia in humans: how breathing pattern modulates heart rate. *Am. J. Physiol.* 241 H620–H629. 7315987 10.1152/ajpheart.1981.241.4.H620

[B29] Iatsenko D. Bernjak A. Stankovski T. Shiogai Y. Owen-Lynch P. J. Clarkson P. B. M. (2013). A. Evolution of cardiorespiratory interactions with age. *Phil. Trans. R. Soc. A* 371:20110622. 10.1098/rsta.2011.0622 23858485 PMC4042892

[B30] Kaminski D. M. Schaan B. D. da Silva A. M. Soares P. P. Lago P. D. (2015). Inspiratory muscle training in patients with diabetic autonomic neuropathy: a randomized clinical trial. *Clin. Auton. Res.* 25 263–266. 10.1007/s10286-015-0291-0 25982993

[B31] Kaplan D. T. Furman I. Pincus S. M. Ryan S. M. Lipsitz L. A. (1991). Aging and the complexity of cardiovascular dynamics. *Biophys. J.* 59 945–949. 10.1016/s0006-3495(91)82309-8 2065195 PMC1281262

[B32] Karsten M. Ribeiro G. S. Esquivel M. S. Matte D. L. (2018). The effects of inspiratory muscle training with linear workload devices on the sports performance and cardiopulmonary function of athletes: a systematic review and meta-analysis. *Phys. Ther. Sport* 34 92–104. 10.1016/j.ptsp.2018.09.004 30261349

[B33] Laitinen T. Hartikainen J. Vanninen E. Niskanen L. Geelen G. Länsimies E. (1998). Age and sex dependency of baroreflex sensitivity in healthy subjects. *J. Appl. Physiol.* 84 576–583. 10.1152/jappl.1998.84.2.576 9475868

[B34] Laitinen T. Niskamen L. Geelen G. Lansimies E. Hartikainen J. (2004). Age dependency of cardiovascular autonomic responses to head-up tilt in healthy subjects. *J. Appl. Physiol.* 96 2333–2340. 10.1152/japplphysiol.00444.2003 14766788

[B35] Lurie K. G. Zielinski T. Voelckel W. McKnite S. Plaisance P. (2002). Augmentation of ventricular preload during treatment of cardiovascular collapse and cardiac arrest. *Crit. Care Med.* 30 S162–S165. 11940794 10.1097/00003246-200204001-00009

[B36] Marchi A. Bari V. De Maria B. Esler M. Lambert E. Baumert M. (2016). Calibrated variability of muscle sympathetic nerve activity during graded head-up tilt in humans and its link with noradrenaline data and cardiovascular rhythms. *Am. J. Physiol.* 310 R1134–R1143. 10.1152/ajpregu.00541.2015 27009053

[B37] Martins de Abreu R. Porta A. Rehder-Santos P. Cairo B. Donisete da Silva C. De Favari Signini E. (2019). Effects of inspiratory muscle training intensity on cardiovascular control in amateur cyclists. *Am. J. Physiol.* 317 R891–R902. 10.1152/ajpregu.00167.2019 31596110

[B38] Martins de Abreu R. Rehder-Santos P. Minatel V. Dos Santos G. L. Catai A. M. (2017). Effects of inspiratory muscle training on cardiovascular autonomic control: a systematic review. *Auton. Neurosci.* 208 29–35. 10.1016/j.autneu.2017.09.002 28916152

[B39] Mazzucco C. E. Marchi A. Bari V. De Maria B. Guzzetti S. Raimondi F. (2017). Mechanical ventilatory modes and cardioventilatory phase synchronization in acute respiratory failure patients. *Physiol. Meas.* 38 895–911. 10.1088/1361-6579/aa56ae 28052047

[B40] Milan-Mattos J. C. Porta A. Perseguini N. M. Minatel V. Rehder-Santos P. Takahashi A. C. M. (2018). Influence of age and gender on the phase and strength of the relation between heart period and systolic blood pressure spontaneous fluctuations. *J. Appl. Physiol.* 124 791–804. 10.1152/japplphysiol.00903.2017 29212671

[B41] Montano N. Gnecchi-Ruscone T. Porta A. Lombardi F. Pagani M. Malliani A. (1994). Power spectrum analysis of heart rate variability to assess changes in sympatho-vagal balance during graded orthostatic tilt. *Circulation* 90 1826–1831. 10.1161/01.cir.90.4.1826 7923668

[B42] Nemati S. Edwards B. A. Lee J. Pittman-Polletta B. Butler J. P. Malhotra A. (2013). Respiration and heart rate complexity: effects of age and gender assessed by band-limited transfer entropy. *Resp. Physiol. Neurobi.* 189 27–33. 10.1016/j.resp.2013.06.016 23811194 PMC3821922

[B43] Nollo G. Faes L. Porta A. Antolini R. Ravelli F. (2005). Exploring directionality in spontaneous heart period and systolic pressure variability interactions in humans: implications in the evaluation of baroreflex gain. *Am. J. Physiol.* 288 H1777–H1785. 15604132 10.1152/ajpheart.00594.2004

[B44] Parati G. di Rienzo M. Bertinieri G. Pomidossi G. Casadei R. Groppelli A. (1988). Evaluation of the baroreceptor-heart rate reflex by 24-hour intra-arterial blood pressure monitoring in humans. *Hypertension* 12 214–222. 10.1161/01.hyp.12.2.214 3410530

[B45] Penzel T. Kantelhardt J. W. Bartsch R. P. Riedl M. Kramer J. Wessel N. (2016). Modulations of heart rate, ECG, and cardio-respiratory coupling observed in polysomnography. *Front. Physiol.* 7:460. 10.3389/fphys.2016.00460 27826247 PMC5078504

[B46] Pomeranz B. Macaulay R. J. B. Caudill M. A. Kutz I. Adam D. Gordon D. (1985). Assessment of autonomic function in humans by heart-rate spectral-analysis. *Am. J. Physiol.* 248 H151–H153. 3970172 10.1152/ajpheart.1985.248.1.H151

[B47] Porta A. Baselli G. Rimoldi O. Malliani A. Pagani M. (2000). Assessing baroreflex gain from spontaneous variability in conscious dogs: role of causality and respiration. *Am. J. Physiol.* 279 H2558–H2567. 11045994 10.1152/ajpheart.2000.279.5.H2558

[B48] Porta A. Bari V. Bassani T. Marchi A. Pistuddi V. Ranucci M. (2013a). Model-based causal closed loop approach to the estimate of baroreflex sensitivity during propofol anesthesia in patients undergoing coronary artery bypass graft. *J. Appl. Physiol.* 115 1032–1042. 10.1152/japplphysiol.00537.2013 23869064

[B49] Porta A. Bassani T. Bari V. Tobaldini E. Takahashi A. C. M. Catai A. M. (2012). Model-based assessment of baroreflex and cardiopulmonary couplings during graded head-up tilt. *Comput. Biol. Med.* 42 298–305.2015. 10.1016/j.compbiomed.2011.04.019 21621756

[B50] Porta A. Castiglioni P. Di Rienzo M. Bassani T. Bari V. Faes L. (2013b). Cardiovascular control and time domain Granger causality: insights from selective autonomic blockade. *Phil. Trans. R. Soc. A* 371:20120161. 10.1098/rsta.2012.0161 23858489

[B51] Porta A. Catai A. M. Takahashi A. C. M. Magagnin V. Bassani T. Tobaldini E. (2011). Causal relationships between heart period and systolic arterial pressure during graded head-up tilt. *Am. J. Physiol.* 300 R378–R386. 10.1152/ajpregu.00553.2010 20962207

[B52] Porta A. Faes L. (2016). Wiener-Granger causality in network physiology with applications to cardiovascular control and neuroscience. *Proc. IEEE* 104 282–309. 10.1109/jproc.2015.2476824

[B53] Porta A. Faes L. Bari V. Marchi A. Bassani T. Nollo G. (2014). Effect of age on complexity and causality of the cardiovascular control: comparison between model-based and model-free approaches. *PLoS One* 9:e89463. 10.1371/journal.pone.0089463 24586796 PMC3933610

[B54] Porta A. Faes L. Nollo G. Bari V. Marchi A. De Maria B. (2015). Conditional self-entropy and conditional joint transfer entropy in heart period variability during graded postural challenge. *PLoS One* 10:e0132851. 10.1371/journal.pone.0132851 26177517 PMC4503559

[B55] Porta A. Maestri R. Bari V. De Maria B. Cairo B. Vaini E. (2018). Paced breathing increases the redundancy of cardiorespiratory control in healthy individuals and chronic heart failure patients. *Entropy* 20:949 10.3390/e20120949 PMC7512533 33266673

[B56] Rehder-Santos P. Minatel V. Milan-Mattos J. C. De Favari Signini E. Abreu R. M. Dato C. C. (2019). Critical inspiratory pressure – a new methodology for evaluating and training the inspiratory musculature for recreational cyclists: study protocol for a randomized controlled trial. *Trials* 20:258. 10.1186/s13063-019-3353-0 31064379 PMC6505302

[B57] Riedl M. Muller A. Kraemer J. F. Penzel T. Kurths J. Wessel N. (2014). Cardio-respiratory coordination increases during sleep apnea. *PLoS One* 9:e93866. 10.1371/journal.pone.0093866 24718564 PMC3981754

[B58] Rodrigues G. D. Gurgel J. L. Gonçalves T. R. da Silva Soares P. P. (2018). Inspiratory muscle training improves physical performance and cardiac autonomic modulation in older women. *Eur. J. Appl. Physiol.* 118 1143–1152. 10.1007/s00421-018-3844-9 29549494

[B59] Saul J. P. Berger R. D. Albrecht P. Stein S. P. Chen M. H. Cohen R. J. (1991). Transfer function analysis of the circulation: unique insights into cardiovascular regulation. *Am. J. Physiol.* 261 H1231–H1245. 1928405 10.1152/ajpheart.1991.261.4.H1231

[B60] Schreiber T. (2000). Measuring information transfer. *Phys. Rev. Lett.* 85 461–464. 10.1103/physrevlett.85.461 10991308

[B61] Schulz S. Adochiei F.-C. Edu I.-R. Schroeder R. Costin H. Bär K.-J. (2013). Cardiovascular and cardiorespiratory coupling analyses: a review. *Phil. Trans. R. Soc. A* 371:20120191. 10.1098/rsta.2012.0191 23858490

[B62] Schulz S. Bär K.-J. Voss A. (2015). Analyses of heart rate, respiration and cardiorespiratory coupling in patients with schizophrenia. *Entropy* 17 483–501. 10.3390/e17020483

[B63] Seals D. R. Suwarno N. O. Dempsey J. A. (1990). Influence of lung volume on sympathetic nerve discharge in normal subjects. *Circ. Res.* 67 130–141. 10.1161/01.res.67.1.130 2364488

[B64] Spyer K. M. (1995). Central nervous mechanisms responsible for cardio-respiratory homeostasis. *Adv. Exp. Med. Biol.* 381 73–79. 10.1007/978-1-4615-1895-2_8 8867825

[B65] Taha B. H. Simon P. M. Dempsey J. A. Skatrud J. B. Iber C. (1995). Respiratory sinus arrhythmia in humans: an obligatory role for vagal feedback from the lungs. *J. Appl. Physiol.* 78 638–645. 10.1152/jappl.1995.78.2.638 7759434

[B66] Task Force (1996). Heart rate variability: standards of measurement, physiological interpretation, and clinical use. Task force of the european society of cardiology and the north american society of pacing and electrophysiology. *Eur. Heart J.* 17 354–381. 8737210

[B67] Taylor J. A. Eckberg D. L. (1996). Fundamental relations between short-term RR interval and arterial pressure oscillations in humans. *Circulation* 93 1527–1532. 10.1161/01.cir.93.8.1527 8608621

[B68] Toska K. Eriksen M. (1993). Respiration-synchronous fluctuations in stroke volume, heart rate and arterial pressure in humans. *J. Physiol.* 472 501–512. 10.1113/jphysiol.1993.sp019958 8145156 PMC1160498

[B69] Vranish J. R. Bailey E. F. (2015). Daily respiratory training with large intrathoracic pressures, but not large lung volumes, lowers blood pressure in normotensive adults. *Respir. Physiol. Neurobiol.* 216 63–69. 10.1016/j.resp.2015.06.002 26112283

[B70] Widjaja D. Montalto A. Vlemincx E. Marinazzo D. Van Huffel S. Faes L. (2015). Cardiorespiratory information dynamics during mental arithmetic and sustained attention. *PLoS One* 10:e0129112. 10.1371/journal.pone.0129112 26042824 PMC4456404

[B71] Yana K. Saul J. P. Berger R. D. Perrott M. H. Cohen R. J. (1993). A time domain approach for the fluctuation analysis of heart rate related to instantaneous lung volume. *IEEE Trans. Biomed. Eng.* 40 74–81. 10.1109/10.204773 8468078

